# BRD7 Promotes Cell Proliferation and Tumor Growth Through Stabilization of c-Myc in Colorectal Cancer

**DOI:** 10.3389/fcell.2021.659392

**Published:** 2021-05-24

**Authors:** Ran Zhao, Yukun Liu, Chunchun Wu, Mengna Li, Yanmei Wei, Weihong Niu, Jing Yang, Songqing Fan, Yong Xie, Hui Li, Wei Wang, Zhaoyang Zeng, Wei Xiong, Xiaoling Li, Guiyuan Li, Ming Zhou

**Affiliations:** ^1^NHC Key Laboratory of Carcinogenesis, Hunan Cancer Hospital and The Affiliated Cancer Hospital of Xiangya School of Medicine, Central South University, Changsha, China; ^2^Cancer Research Institute and School of Basic Medical Sciences, Central South University, Changsha, China; ^3^Department of Pathology, Affiliated Hospital of Jining Medical University, Jining Medical University, Jining, China; ^4^The Key Laboratory of Carcinogenesis and Cancer Invasion of the Chinese Ministry of Education, Central South University, Changsha, China; ^5^Department of Pathology, The Second Xiangya Hospital, Central South University, Changsha, China; ^6^Hunan Key Laboratory of Oncotarget Gene, Hunan Cancer Hospital and The Affiliated Cancer Hospital of Xiangya School of Medicine, Central South University, Changsha, China

**Keywords:** BRD7, oncogene, c-Myc, deubiquitination, colorectal cancer

## Abstract

BRD7 functions as a crucial tumor suppressor in numerous malignancies. However, the effects of BRD7 on colorectal cancer (CRC) progression are still unknown. Here, based on the BRD7 knockout (BRD7^–/–^) and BRD7^*flox*/*flox*^ (BRD7^+/+^) mouse models constructed in our previous work, we established an azoxymethane/dextran sodium sulfate (AOM/DSS)-induced mouse model. BRD7^+/+^ mice were found to be highly susceptible to AOM/DSS-induced colitis-associated CRC, and BRD7 significantly promoted cell proliferation and cell cycle G1/S transition but showed no significant effect on cell apoptosis. Furthermore, BRD7 interacted with c-Myc and stabilized c-Myc by inhibiting its ubiquitin–proteasome-dependent degradation. Moreover, restoring the expression of c-Myc in BRD7-silenced CRC cells restored cell proliferation, cell cycle progression, and tumor growth *in vitro* and *in vivo*. In addition, BRD7 and c-Myc were both significantly upregulated in CRC patients, and high expression of these proteins was associated with clinical stage and poor prognosis in CRC patients. Collectively, BRD7 functions as an oncogene and promotes CRC progression by regulating the ubiquitin–proteasome-dependent stabilization of c-Myc protein. Targeting the BRD7/c-Myc axis could be a potential therapeutic strategy for CRC.

## Introduction

Colorectal cancer (CRC) is the third most common gastrointestinal cancer and the fourth most deadly cancer worldwide (Dekker et al., 2019). Over the past decade, CRC incidence has continued to decline by approximately 2% per year, which is largely attributed to premalignant lesion excision and the practice of colonoscopy (Saunders and Tsiamoulos, 2016; Wolf et al., 2018; Siegel et al., 2019). However, owing to the complicated pathological mechanisms of CRC initiation and progression, declines in mortality from CRC have markedly slowed during recent years (Arnold et al., 2017). Therefore, it is necessary to further clarify the mechanism of pathogenesis of CRC and identify sensitive and specific biomarkers and potential therapeutic targets for the diagnosis and treatment of CRC.

c-Myc functions as a key transcription factor and is involved in the regulation of over 15% genes in the human transcriptome (Dang et al., 2006; Kress et al., 2015), which affects almost all cellular processes and is essential for normal cellular homeostasis (Scognamiglio et al., 2016; Perez-Olivares et al., 2018; Sanchez-Arevalo Lobo et al., 2018). Under physiological conditions, c-Myc protein stability and activity are tightly controlled via the ubiquitin–proteasome system (Li et al., 2015; Sun et al., 2015). In normal cells, c-Myc is an extremely unstable protein with a half-life of 20–30 min, and its rapid turnover is triggered primarily by the posttranslational modification of the T58 and S62 phosphorylation (Wang W. et al., 2016; Dingar et al., 2018). Increasing evidence has shown that enhanced stabilization of the c-Myc protein contributes to its ectopic expression and deregulated control of cell proliferation and, subsequently, cancer initiation and development (Zhang P. et al., 2016; Zhang et al., 2018). Consistently, c-Myc is aberrantly overexpressed or hyperactivated in more than 50% of malignancies and exerts its pro-oncogenic effects through accelerating cell cycle progression, promoting metastasis, and reprogramming metabolism (Zwolinska et al., 2012).

BRD7 is a ubiquitously expressed bromodomain-containing protein, which generally interacts with numerous chromatin remodeling factors and transcription factors (Drost et al., 2010; Harte et al., 2010; Pan et al., 2018). Consequently, BRD7 plays crucial roles in many aspects of cellular processes, such as cell proliferation, apoptosis, differentiation, and glucose metabolism (Zhou et al., 2004; Park S. W. et al., 2014; Wang et al., 2017; Liu et al., 2019); and its abnormal expression is broadly associated with the development of many diseases (Xu Y. et al., 2015; Wang H. et al., 2016; Zhao et al., 2017; Wei et al., 2018), including cancers. To date, considerable evidence has shown that BRD7 is commonly downregulated in many malignancies, such as nasopharyngeal carcinoma, epithelial ovarian cancer, and breast cancer, and BRD7 functions as a tumor suppressor (Park Y. A. et al., 2014; Liu et al., 2018; Ma et al., 2019). In our previous studies, we found that BRD7 inhibits cell proliferation and tumor growth by maintaining cell cycle G1/S phase arrest in nasopharyngeal carcinoma cells (Zhou et al., 2004; Liu et al., 2016, 2018). Moreover, consistent with its tumor-suppressive role, BRD7 exerts an anti-inflammatory effect in the early stage of dextran sodium sulfate (DSS)-induced colitis in a BRD7 knockout mouse model (Zhao et al., 2017). However, the functional role and mechanism of BRD7 in CRC initiation and development are still unknown and need to be further studied.

In this study, we further employed a well-established azoxymethane (AOM)/DSS model of colitis-associated cancer with BRD7 knockout mice and determined the functional role of BRD7 in colitis-associated CRC. Subsequently, we confirmed the tumor-promoting properties of BRD7, including cell proliferation, cell cycle progression, and tumor growth, *in vitro* and *in vivo*. Moreover, we determined the structural basis of the interaction between BRD7 and c-Myc and the impact of BRD7 on the ubiquitin–proteasome-mediated degradation of c-Myc protein. Furthermore, we explored the role of the BRD7/c-Myc axis in cell proliferation *in vitro* and *in vivo* and the clinical significance of BRD7 and c-Myc in CRC patients. Our study demonstrated that BRD7 promotes CRC progression through stabilizing the c-Myc protein and protecting it from ubiquitin–proteasome-mediated degradation and that BRD7 functions as a promising prognostic factor and a potential therapeutic target for CRC.

## Materials and Methods

### Ethics Statement

Ethical approval for this study was obtained from the Ethics Review Committees/Institutional Review Boards of Central South University and Affiliated Hospital of Jining Medical University. All *in vivo* procedures were conducted in accordance with protocols approved by the Institutional Animal Care and Use Committee (IACUC) of Central South University.

### Human Colorectal Cancer Tissues

Human CRC samples were collected at the Second Xiangya Hospital of Central South University (Changsha, China) and the Affiliated Hospital of Jining Medical University (Shandong, China). Written informed consent was obtained from all patients. Clinical data are shown in Table 1. All of the 180 patients had valid follow-up data. Overall survival (OS) was defined as the time from diagnosis to the date of death or the date that the patient was last known to be alive. All specimens were confirmed by pathological diagnosis according to the criteria of the American Joint Committee on Cancer (AJCC, 8th edition).

**TABLE 1:** Association between the expression of BRD7, c-Myc, and CRC clinical pathological features (*N* = 180).

Characteristics (N)	BRD7			c-Myc			BRDT/c-Myc^a^		
			
	H (%)	L (%)	*P*	H (%)	L (%)	*P*	H–H (%)	L–L (%)	*P*
Age (year)									
<60 (*n* = 74)	41 (55.4)	33 (44.6)	0.642	37 (50.0)	37 (50.0)	0.533	22 (29.7)	18 (24.3)	0.465
≥60 (*n* = 106)	55 (51.9)	51 (48.1)		48 (45.3)	58 (54.7)		30 (28.3)	33 (31.1)	
Gender									
Female (*n* = 82)	46 (56.1)	36 (43.9)	0.497	45 (54.9)	37 (45.1)	0.060	30 (36.6)	21 (25.6)	0.094
Male (*n* = 98)	50 (51.0)	48 (49.0)		40 (40.8)	58 (59.2)		22 (22.4)	30 (30.6)	
Differentiation grade									
Well (*n* = 5)	0 (0.0)	5 (100.0)	<0.0001*	2 (40.0)	3 (60.0)	1.000	0 (0.0)	3 (60.0)	0.0005*
Moderately (*n* = 90)	33 (36.7)	57 (63.3)		44 (48.9)	46 (51.1)		18 (20.0)	31 (34.4)	
Poorly (*n* = 85)	63 (74.1)	22 (25.9)		39 (45.9)	46 (54.1)		34 (40.0)	17 (20.0)	
Clinical stages			0.024*			0.833			0.027*
Stage I–II (*n* = 129)	62 (48.1)	67 (51.9)		58 (45.0)	71 (55.0)		34 (26.4)	43 (33.3)	
Stage III–IV (*n* = 51)	34 (66.7)	17 (33.3)		27 (52.9)	24 (47.1)		18 (35.3)	8 (15.7)	

*H, high expression; L, low expression.*

^a^H–H, High expression of BRD7 and high expression of c-Myc; L–L, low expression of BRD7 and low expression of c-Myc; CRC, colorectal cancer. *P < 0.05.

### Cell Lines and Culture

HEK293, HEK293T, NCM460, HCT116, SW480, SW620, and HT29 cell lines were preserved in our laboratory. HEK293 and HEK293T cells were cultured in Dulbecco’s modified Eagle medium (DMEM) supplemented with 10% fetal bovine serum (FBS) and antibiotics including 1% penicillin and 1% streptomycin (all purchased from Gibco, Invitrogen, Paisley, United Kingdom). The other cell lines were cultured in RPMI-1640 with 10% FBS and antibiotics (Gibco) at 37°C in a 5% CO_2_, humidified incubator.

### Azoxymethane/Dextran Sodium Sulfate-Induced Colitis-Associated Carcinogenesis Mouse Model

**FIGURE 1:**
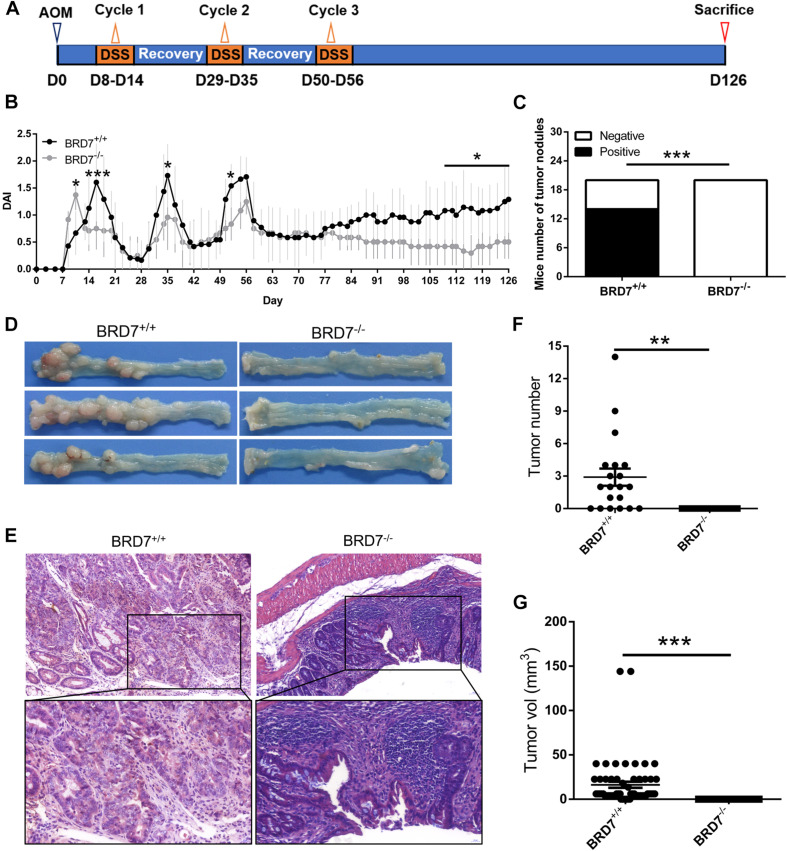
BRD7 promotes azoxymethane/dextran sodium sulfate (AOM/DSS)-induced colitis-associated carcinogenesis in mice. **(A)** Schematic representation of the AOM/DSS-induced colitis-associated colorectal cancer (CRC) protocol. **(B)** disease activity index (DAI) score of BRD7^+/+^ and BRD7^–/–^ mice following AOM/DSS treatment, *n* = 20. **(C)** The number of BRD7^+/+^ and BRD7^–/–^ mice harboring tumor nodules. **(D)** Representative images of the colorectum of BRD7^+/+^ and BRD7^–/–^ mice on day 126. **(E)** Hematoxylin and eosin (H&E) staining of colorectal tissues of BRD7^+/+^ and BRD7^–/–^ mice. Images in the top panel were representative of H&E staining. Scale bar: 50 μm. Images in the bottom panel were magnified from the black box area in the images in the top panel, respectively. Scale bar: 20 μm. **(F)** The number and **(G)** volume of colorectal tumors in BRD7^+/+^ and BRD7^–/–^ mice. The results are presented as the mean ± SD. *P < 0.05, **P < 0.01, ***P < 0.001.

BRD7 knockout (BRD7^–/–^) mouse model was constructed in our previous publication (Wang H. et al., 2016; Zhao et al., 2017). BRD7^*flox*/*flox*^ (BRD7^+/+^) (*n* = 20) or BRD7 knockout (BRD7^–/–^) (*n* = 20) C57BL/6 mice at 8–12 weeks old were injected intraperitoneally with 10 mg/kg of AOM (Sigma-Aldrich, St. Louis, MO, United States). After a recovery week, mice were administered 2% DSS (molecular weight, 36,000–50,000; MP Biomedicals, Solon, OH, United States) in their drinking water for 1 week, followed by another 2 weeks of recovery. This cycle was repeated three times. After the last cycle, mice were euthanized after an additional 8-week recovery period (Schematic in Figure 1A). The number and volume of colon lesions were counted and measured, respectively, using a stereomicroscope.

The disease activity index (DAI) score was described in our previous report (Zhao et al., 2017). In brief, the body weight, stool consistency, and fecal blood of mice were monitored daily, and the DAI was calculated according to a modified protocol by grading weight loss on a scale of 0–4 (0 = normal; 1 = 1–5%; 2 = 5–10%; 3 = 10–20%; 4 ≥ 20%), stool consistency on a scale of 0–2 (0 = normal; 1 = loose stools; 2 = diarrhea), and the presence of fecal blood on a scale of 0–2 (0 = normal; 1 = bleeding; 2 = severe bleeding). The final DAI value for each mouse was expressed as the average of the combined scores.

For histological analysis, the colon tissues resembling a “Swiss roll” were fixed in 4% paraformaldehyde and embedded in paraffin. Fixed tissues were cut into 4-μm-thick sections, placed on glass slides, and deparaffinized. The sections were stained with hematoxylin and eosin (H&E) and observed under a light microscope to confirm the severity and grade of dysplasia.

### Plasmids and Domain Deletion Mutants of BRD7 and c-Myc

Full-length GFP-tagged BRD7 (pEGFP-C2/BRD7), Flag-tagged BRD7 (pIRESneo3/3Flag-BRD7), HA-tagged BRD7 (pCMV-HA-BRD7), and Flag-tagged c-Myc (pIRESneo3/2Flag-c-Myc) expression recombinant vectors and truncated mutants of BRD7 (pCMV-HA-N-Ter, HA-C-Ter, HA-ΔN-Ter, HA-ΔBRD, and HA-ΔC-Ter) and c-Myc (Flag-TAD, Flag-CP, and Flag-bHLHZ) were constructed by our laboratory. The structures of BRD7 and c-Myc truncated mutants are shown in Figure 4.

### Transfection and Stable Cell Lines

To generate stable BRD7-overexpressing cells, HCT116 and SW620 cells were transfected with pIRESneo3/3Flag-BRD7 or empty plasmid using Lipofectamine 3000 (Invitrogen, Carlsbad, CA, United States) according to the manufacturer’s protocol. The plasmid encoding the full-length open reading frame (ORF) of human BRD7 with 3Flag tags (pIRESneo3/3Flag-BRD7) was constructed by our laboratory. The stably transfected cells were selected with G418 (Invitrogen) for 2 weeks. Stable pool clones were obtained.

Two siRNAs (siBRD7#1 and siBRD7#2) and negative control siRNA (NControl, NControl_05815) were purchased from RiboBio (Guangdong, China). For BRD7 knockdown, we transiently transfected HCT116 and SW620 cells with siRNAs or NControl using Lipofectamine 3000 (Invitrogen) according to the manufacturer’s protocol. The sequences of siBRD7#1 and siBRD7#2 were 5′-AUACGGAUAAUCUUGGCACdTdT-3′ and 5′-UAAAUCAUGGCAUUAGUACdTdT-3′, respectively.

To generate stable BRD7 knockdown cell lines, we further selected the efficient siRNA (siBRD7#2) and purchased lentiviral vectors that included the shRNA targeting BRD7 (GV115/shBRD7) and shRNA control (shControl) from GeneChem (Shanghai, China). HCT116 and SW620 cells were infected with the BRD7 shRNA lentiviral vector or shControl vector. Subsequently, the infected cells were screened using fluorescence-activated cell sorting with flow cytometry (BD Biosciences, MD, United States) to obtain a stable pool of clones.

### RNA Extraction, qPCR, and Reverse Transcription-PCR

Total RNA was extracted from the cultured cells using TRIzol (Invitrogen). cDNA was synthesized from the total RNA using a reverse transcription system (Fermentas, Glen Burnie, MD, United States) according to the manufacturer’s instructions. GAPDH was amplified in parallel as an internal control. The expression level of each gene was quantified by measuring the cycle threshold (Ct) values, which were normalized relative to those of GAPDH using the 2^–ΔΔCt^ method. PCR was performed using PCR Mix (BioTeke, Beijing, China). The PCR products were analyzed by electrophoresis through a 1.5% agarose gel. Primer pair sequences for BRD7 were 5′-GCTGTTGCACTCAGGAATGA-3′ (forward) and 5′-ACTCTTGAAGGCGTGTGCTT-3′ (reverse). Primer pair sequences for GAPDH were 5′-CGAGATCCCTCCAAAAT CAA-3′ (forward) and 5′-TTCACACCCATGACGAACAT-3′ (reverse). PCRs of each sample were conducted in triplicate.

### Cell Proliferation (Cell Counting Kit-8), 5-Ethynyl-2’-Deoxyuridine Incorporation, and Colony Formation Assays

Cells were seeded onto 96-well plates (1,000 cells/well). A cell growth curve was constructed using a Cell Counting Kit-8 (CCK-8; Bimake, Houston, TX, United States). Absorbance was measured at 450 nm using a microplate spectrometer. For EdU cell proliferation assays, EdU incorporation assays were performed using a Cell-Light EdU Apollo567 *in vitro* Kit (RiboBio, Guangzhou, China) according to the manufacturer’s protocol.

For colony formation, 500 cells were seeded into a 6-well plate, with three wells per sample. After a 14-day incubation, the cells were fixed with 4% paraformaldehyde and stained with Giemsa solution (Beyotime, Beijing, China). Colonies containing more than 50 cells were counted as 1 positive colony. The plate clone formation efficiency was calculated as (number of colonies/number of cells inoculated) × 100%. All experiments were performed in triplicate.

### Flow Cytometry for Cell Cycle and Apoptosis

The cell cycle and apoptosis analysis was described in our previous work (Liu et al., 2016). Briefly, cells were harvested and fixed in 70% ethanol for 24 h at -20°C, then treated with RNase A, and stained with 25 μg/ml of propidium iodide (PI) (Beyotime). For cell apoptosis analysis, the ratio of apoptotic cells was determined using an Annexin V-PE/7-AAD double staining kit (BD). Samples were all analyzed using a MoFlo XDP High-Performance Cell Sorter (Beckman Coulter, CA, United States), and the data were analyzed using Summit v.5.2 software according to the manufacturer’s protocol. At least three independent experiments were performed.

### Western Blot Analysis

Briefly, cells were lysed in radioimmunoprecipitation assay (RIPA) buffer containing both Protease Inhibitor Cocktail and PhoSTOP (Roche, Basel, Switzerland). The total proteins were quantified using the BCA Protein Assay Kit (Pierce Biotechnology, Rockford, IL, United States), separated by sodium dodecyl sulfate–polyacrylamide gel (SDS-PAGE) electrophoresis, and transferred onto polyvinylidene fluoride (PVDF) membranes (Millipore, Billerica, MA, United States). The membranes were blocked with 5% non-fat milk in Tris-buffered saline and then incubated with primary antibodies at 4°C overnight. The primary antibodies used were anti-BRD7 (dilution 1:500; ProteinTech, Wuhan, China), anti-c-Myc (dilution 1:1,000; CST, Danvers, MA, United States), anti-Flag (dilution 1:2,000; Sigma, MO, United States), anti-HA (dilution 1:2,000; ProteinTech), anti-GAPDH (dilution 1:5,000; ProteinTech), anti-Phospho-c-Myc (Thr58) (dilution 1:1,000; CST), anti-CDC25A (dilution 1:500; ProteinTech), anti-CCND1 (dilution 1:500; Santa Cruz), and anti-CDK4 (dilution 1:500; Santa Cruz). The membranes were then washed three times in TBST solution for 10 min each time and then incubated with secondary antibodies. Signals were detected with an enhanced chemiluminescence detection system (Bio-Rad, Hercules, CA, United States).

### Deubiquitination Assay of c-Myc Protein

BRD7-overexpressing HCT116 cells or control cells were treated with the proteasome inhibitor MG132 (Selleck, Houston, United States) for 3 h. The cells were lysed with immunoprecipitation (IP) buffer and then were sonicated, and the lysates were centrifuged. The supernatant was subjected to IP assay with anti-c-Myc (CST). Subsequently, the sample was subjected to western blotting with an anti-ubiquitin antibody (CST).

### Immunofluorescence and Co-Immunoprecipitation

For immunofluorescence (IF), BRD7-overexpressing HEK293 and HCT116 cells were seeded on glass coverslips, fixed with 4% paraformaldehyde for 15 min at room temperature, and then permeabilized with 0.3% Triton X-100 for 5 min at room temperature. Cells were washed three times with TBS, incubated for 15 min in 5% goat serum, and then incubated at 4°C overnight with the anti-c-Myc primary antibody (dilution 1:800; CST). After three 5-min washes with TBS, cells were incubated for 1 h at 37°C in the dark with the Alexa Fluor 568 donkey anti-Rabbit IgG (H + L) secondary antibody (dilution 1:1,000; Invitrogen). Coverslips were washed with TBS and visualized using an epifluorescence microscope.

For Co-IP, HEK293T or HCT116 cells were transfected with pIRESneo3/2Flag-c-Myc or pCMV-HA-BRD7 expression vector and their truncated mutants together, respectively. Cells were lysed with cell lysis buffer for western blotting and IP (Beyotime, Beijing, China). They were then premixed with protease inhibitor cocktail (Roche) on ice for 20 min and centrifuged at 12,000 *g* for 15 min. The supernatants were incubated at 4°C overnight with 30 μl of anti-Flag or anti-HA magnetic beads (Biomake) and anti-mouse IgG (Santa Cruz). The immunocomplexes were isolated and purified according to the manufacturer’s protocol and then subjected to western blot analysis.

### HCT116 and SW620 Tumor Xenograft Model

A total volume of 100 μl of BRD7 stable knockdown HCT116 and SW620 cells or control cells (5 × 10^6^ cells) was inoculated subcutaneously into the right flanks of 6-week-old female nude mice. Mice were checked every 4 days, and tumor nodules were measured with a caliper. Tumor volume was evaluated as described in our previous work (Liu et al., 2016). Tumor growth curves were calculated. Mice in the three experimental groups were killed after 24 days. All tumors were excised, weighed, harvested, fixed, and embedded in paraffin. The anti-Ki-67 antibody (dilution 1:100; Bioworld Technology, Atlanta, GA, United States) was used to detect the proliferation marker Ki-67 using immunohistochemistry (IHC) procedures. Samples were observed using an Olympus microscope (Olympus, Tokyo, Japan).

### Immunohistochemistry Staining and Scores

IHC staining was described in our previous work (Zhao et al., 2017). Briefly, the sections were rehydrated, and antigen retrieval was performed by boiling in 0.01 M of citrate buffer (pH 6.0). After inhibition of endogenous peroxidase activity, the sections were blocked with 2% bovine serum albumin in phosphate-buffered saline (PBS) and then incubated with anti-BRD7 (dilution 1:100; ProteinTech), anti-c-Myc (dilution 1:100; Millipore), anti-CDK4 (dilution 1:100; Santa Cruz), anti-CCND1 (dilution 1:100; Santa Cruz), and anti-CDC25A (dilution 1:100; ProteinTech) antibodies. The immune complex was visualized with the MaxVison HRP-polymer IHC Kit Detection System, Peroxidase/DAB, Rabbit/Mouse (MaxVison, Fuzhou, China) according to the manufacturer’s protocol. The nuclei were counterstained with hematoxylin (Beyotime). Positive control slides were included in every experiment in addition to the internal positive control. The specificity of the antibody was determined using a matched IgG isotype antibody as a negative control.

For IHC scores, IHC staining was evaluated at 200 × magnification using light microscopy and independently assessed by two pathologists who were blinded to the clinicopathological data. The score of BRD7 and c-Myc protein in nucleus compartment was calculated according to the staining intensity and extent of staining using a method described in our previous work (Liu et al., 2018).

### Statistical Analysis

The correlation between the expression of BRD7 and c-Myc protein and the clinicopathological characteristics of CRC patients were evaluated using the chi-square test. Spearman’s rank correlation coefficient was used to assess the significance of the association between the expression of BRD7 and c-Myc in CRC. Kaplan–Meier analysis was performed to generate OS curves, and statistical significance was assessed using the log-rank test. The differences between the groups were analyzed using Student’s *t*-test when there were only two groups or using one-way ANOVA when there were more than two groups. All statistical analyses were performed using SPSS software (SPSS, Chicago, IL, United States). A two-tailed value of *P* < 0.05 was considered statistically significant.

## Results

### Knockout of BRD7 Inhibits Tumorigenesis and Tumor Growth in Azoxymethane/Dextran Sodium Sulfate-Induced Colorectal Cancer Model

Previous studies have reported that BRD7 is aberrantly of low levels of expression in many malignancies and significantly associated with malignant features and poor prognosis (Park Y. A. et al., 2014; Liu et al., 2018; Ma et al., 2019). To explore the functional role of BRD7 in CRC progression, we investigated the effect of BRD7 knockout on AOM/DSS-induced colitis-associated carcinogenesis (CAC) in mice. BRD7^+/+^ and BRD7^–/–^ mice were injected with AOM and then subjected to three rounds of 2% DSS exposure to induce CRC (Figure 1A). As a result, BRD7^–/–^ mice show an increased DAI before the 14th day than did BRD7^+/+^ mice, which is consistent with our previous results (Zhao et al., 2017), but this was followed by a quick reduction in the DAI until the mice were euthanized (Figure 1B); these results demonstrated that BRD7^+/+^ mice suffered more severe colitis after the 14th day, suggesting that the deficiency of BRD7 plays a protective role in the middle-late stage of CAC. More surprisingly, 70% of BRD7^+/+^ mice displayed tumor nodules in the colorectum, while no tumor nodules were observed in the BRD7^–/–^ group (Figures 1C,D). Furthermore, we performed H&E staining and found that BRD7^–/–^ mice developed low-grade dysplasia, while BRD7^+/+^ mice displayed high-grade dysplasia characterized by loss of epithelial polarity (Figure 1E) and an increased tumor multiplicity in the colons, measured as the sum of tumor numbers and tumor volume, respectively (Figures 1F,G). To exclude the genetic inactivation caused by chemical-induced mutations, we detected the ORF sequences of BRD7 in tumors of BRD7^+/+^ mice and confirmed that there were no mutants in the BRD7 ORF as shown in Supplementary Figure 1, which suggest that the mechanism that BRD7 promotes tumor development and that growth is not dependent on the loss of a tumor-suppressive role resulted from AOM/DSS-driven BRD7 mutation but exerts oncogenic roles. Taken together, all of the findings above revealed that BRD7^+/+^ mice are highly susceptible to colitis-associated CRC and that BRD7 plays a crucial cancer-promoting role in colorectal carcinogenesis in mice.

### BRD7 Promotes Cell Proliferation by Accelerating the G1/S Phase Transition in Human Colorectal Cancer Cells

**FIGURE 2:**
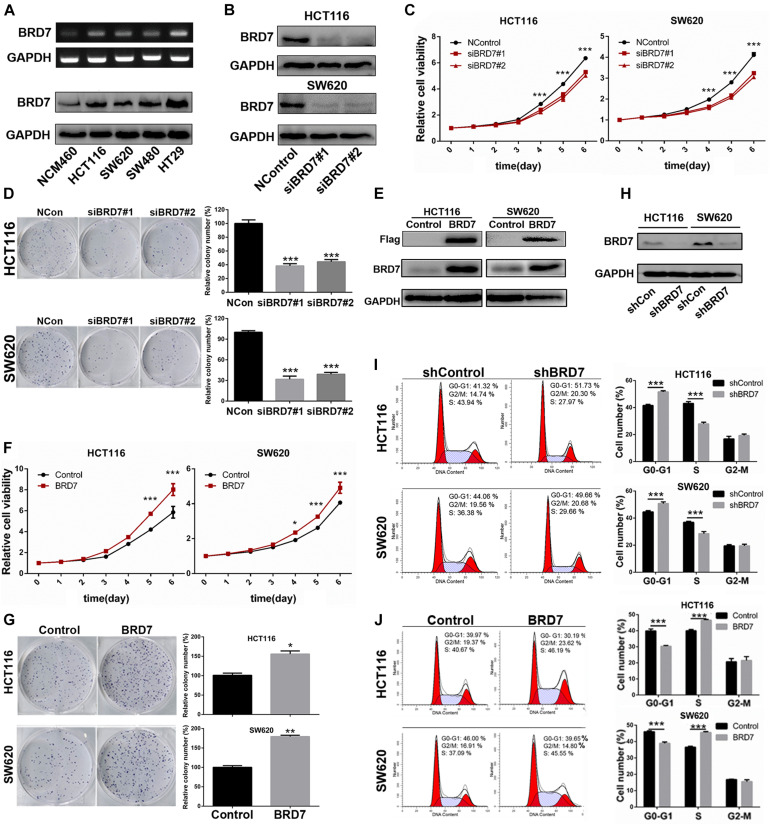
Effect of BRD7 on the cell proliferation and cell cycle of colorectal cancer (CRC) cells. **(A)** BRD7 mRNA and protein expression levels were detected by RT-PCR and western blotting in HCT116, SW620, SW480, and HT29 CRC cell lines and the normal colon mucosal epithelial cell line NCM460. **(B)** Western blotting confirmed the interference efficiency of BRD7 siRNAs on endogenous BRD7 protein levels in HCT116 and SW620 cells. **(C,F)** The effect of BRD7 knockdown and overexpression on cell viability detected by CCK-8 assays. **(D,G)** The effect of BRD7 knockdown and overexpression on the colony formation of HCT116 and SW620 cells. **(E,H)** Western blot analysis confirmed the BRD7 protein expression levels in HCT116 and SW620 cells with stable BRD7 overexpressing or knockdown, respectively. **(I,J)** The effect of BRD7 stable knockdown or overexpression on cell cycle progression, as determined by flow cytometry. The results are presented as the mean ± SD. *P < 0.05, **P < 0.01, ***P < 0.001.

Then, we quantified the expression of BRD7 in the human CRC cell lines HCT116, SW620, SW480, and HT29 and the normal colon mucosal epithelial cell line NCM460. The RT-PCR and western blotting results showed that the mRNA and protein expression of BRD7 were both markedly upregulated in CRC cell lines when compared with NCM460 (Figure 2A), and HCT116 and SW620 cells were therefore chosen as the cell models for investigation of BRD7 function and mechanism based on their moderate expression among these CRC cell lines. To further confirm the role of BRD7 in CRC progression, we transiently transfected HCT116 and SW620 cell lines with BRD7-siRNAs (siBRD7#1 and siBRD7#2) and determined the endogenous levels of BRD7 mRNA and protein, respectively. The results showed that the levels of endogenous BRD7 expression were significantly downregulated in both HCT116 and SW620 cell lines (Supplementary Figure 2A and Figure 2B). Subsequently, we performed CCK-8 and colony forming assays to determine the effect of BRD7 knockdown on cell proliferation, and the results showed that BRD7 knockdown significantly inhibited cell proliferation and colony formation in both HCT116 and SW620 cell lines (Figures 2C,D). In contrast, ectopic expression of BRD7 markedly promoted cell proliferation in the two cell lines (Figures 2E–G).

To further illuminate the cell proliferation-promoting role of BRD7 in CRC cells, we further constructed BRD7 stable knockdown HCT116 and SW620 cell lines based on the most efficient siRNA (siBRD7#2) (Supplementary Figure 2B and Figure 2H) and examined the effect of stable BRD7 knockdown and overexpression on cell cycle progression in these two cell lines. The results showed that BRD7 knockdown caused significant G0/G1 phase arrest (Figure 2I), while ectopic expression of BRD7 markedly promoted G1/S phase transition (Figure 2J). Furthermore, we performed EdU incorporation assays to confirm the role of BRD7 in promoting cell cycle progression, and the results showed that BRD7 knockdown significantly reduced EdU incorporation in both HCT116 and SW620 cell lines (Supplementary Figure 2C) and that BRD7 overexpression played the opposite role (Supplementary Figure 2D). In addition, BRD7 knockdown or overexpression had no significant effect on cell apoptosis in either HCT116 or SW620 cells (Supplementary Figures 2E,F). Taken together, these results demonstrated that BRD7 functions as a promoting factor in CRC cell proliferation by accelerating the G1/S phase transition of the cell cycle.

### BRD7 Increases the Stability of c-Myc Protein by Decreasing the Ubiquitination of c-Myc

**FIGURE 3:**
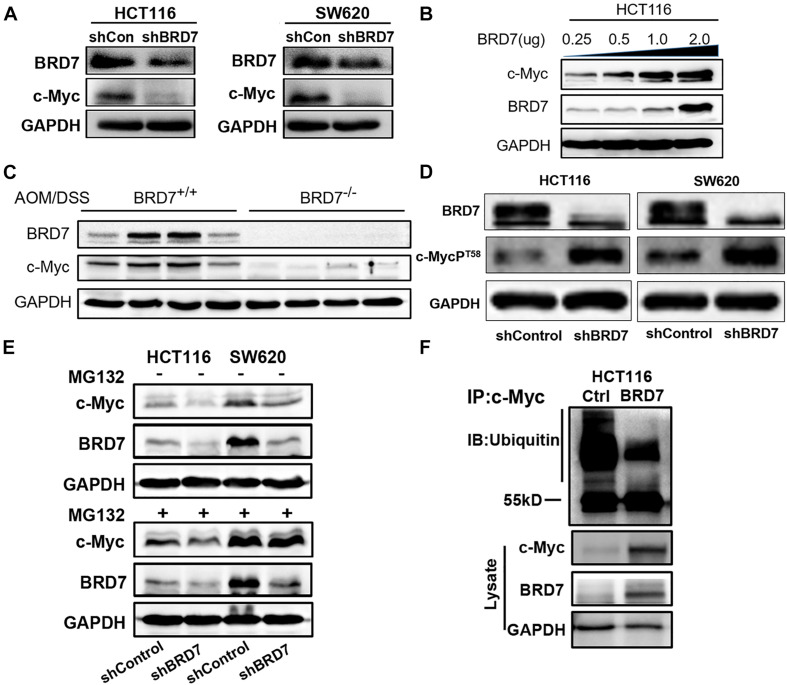
BRD7 enhances the stability of the c-Myc protein by inhibiting its ubiquitination. **(A)** Western blot analysis detected the effect of BRD7 knockdown on c-Myc protein expression in HCT116 and SW620 cell lines. **(B)** The effect of BRD7 overexpression on c-Myc protein in HCT116 cells at different doses. **(C)** The expression of BRD7 and c-Myc protein in the colorectal tissues of azoxymethane/dextran sodium sulfate (AOM/DSS)-induced colorectal cancer (CRC) mice (*N* = 4). **(D)** Western blot analysis showed the effect of BRD7 knockdown on the phosphorylation levels of c-Myc protein at T58 sites in both HCT116 and SW620 cells. **(E)** Western blot analysis showed the effect of BRD7 knockdown on ubiquitin–proteasome-mediated protein stability of c-Myc in MG132-treated HCT116 and SW620 cells. MG132, proteasome inhibitor MG132. **(F)** Immunoprecipitation (IP) and western blot assays confirmed the effect of BRD7 on the ubiquitination of c-Myc protein.

As c-Myc is aberrantly overexpressed or hyperactivated and exerts its pro-oncogenic roles in more than 50% of malignancies (Zwolinska et al., 2012), including CRC, we detected the effect of BRD7 on c-Myc expression. The expression of c-Myc protein was significantly downregulated in BRD7 knockdown HCT116 and SW620 cells (Figure 3A), and the half-life of c-Myc protein was markedly reduced by BRD7 knockdown (Supplementary Figure 3). Accordingly, ectopic expression of BRD7 increased c-Myc protein expression in a dose-dependent manner (Figure 3B), while there were no significant changes in the mRNA level of c-Myc (Supplementary Figure 4). In addition, the expression of c-Myc protein was significantly upregulated in BRD7^+/+^ group than the BRD7^–/–^ group of AOM/DSS-induced CRC model (Figure 3C). These results indicate that BRD7 might be involved in the regulation of c-Myc stability. As c-Myc T58 phosphorylation is tightly correlated to the degradation of c-Myc through the proteasome system (Sun et al., 2015; Wang W. et al., 2016), we therefore detected the phosphorylation level of c-Myc protein, and the immunoblotting results showed that BRD7 knockdown significantly increased the c-Myc T58 phosphorylation levels (Figure 3D). To further determine the effect of BRD7 on c-Myc protein stability, we explored the functional role of BRD7 in c-Myc protein degradation mediated by ubiquitin–proteasome. As a result, knockdown of BRD7 decreased the protein level of c-Myc consistent with the previous results, while c-Myc protein degradation was obviously hindered after the cells were treated with proteasome inhibitor, MG132, in both BRD7 knockdown HCT116 and SW620 cells (Figure 3E). Moreover, ectopic expression of BRD7 significantly reduced the level of c-Myc ubiquitination (Figure 3F). Taken together, our findings demonstrated that BRD7 can stabilize c-Myc protein by inhibiting its ubiquitination dependent on its phosphorylation at the T58 site. In addition, we examined whether FBW7 mediates the degradation of c-Myc after BRD7 knockdown. We found that BRD7 knockdown decreases c-Myc protein level, which is consistent with the previous results, but when FBW7 was depleted in BRD7 knockdown HCT116 cells, the stability of c-Myc is clearly recovered (Supplementary Figure 5). The results suggested that FBW7 is involved in the stabilization of c-Myc mediated by BRD7.

### BRD7 Interacts With c-Myc in a Manner Dependent on the N-Terminus of BRD7 and the CP-Domain of c-Myc

**FIGURE 4:**
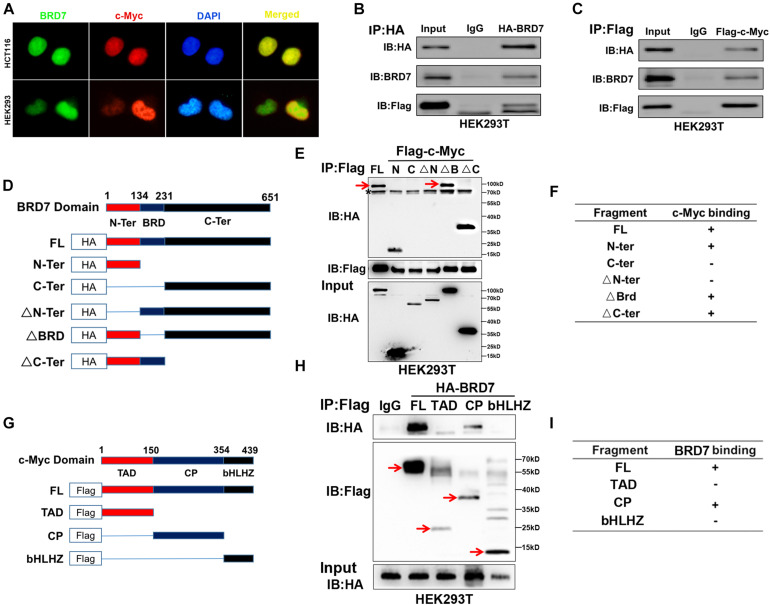
BRD7 interacts with c-Myc in a manner dependent on the N-terminus of BRD7 and the CP-domain of c-Myc. **(A)** Immunofluorescence assays showed the localization of BRD7 and c-Myc in HCT116 and HEK293 cells. **(B,C)** Co-immunoprecipitation (Co-IP) confirmed the interaction between BRD7 and c-Myc in HEK293T cells. **(D,G)** The schematic diagram of different truncated mutants of BRD7 and c-Myc domains. **(E,F,H,I)** Co-IP assays revealed the structural basis of the interaction between BRD7 and c-Myc. The antibody used for IP detection in **(E,H)** is anti-Flag antibody, and the antibodies used for IB detection are anti-HA and anti-Flag antibodies, respectively. Of them, Flag was used to tag c-Myc and its mutants, while HA was used to tag BRD7 and its mutants. FL, full length; TAD, transcription activation domain; CP, central portion flanked by TAD and bHLHZ; bHLHZ, basic helix-loop-helix leucine zipper; N-Ter/N, N-terminal; C-Ter/C, C-terminal; ΔN-Ter/ΔN, N-terminal deletion; ΔBRD, bromodomain deletion; ΔC-Ter/ΔC, C-terminal deletion. The arrow indicates the destination bands, and * indicates non-specific bands.

Since BRD7 increased c-Myc protein stability through the ubiquitin–proteasome pathway, we investigated whether they interacted with each other. IF and nuclear/cytosol fractionation assays showed that both BRD7 and c-Myc were predominantly colocalized in the nucleus in both HEK293 and HCT116 cells (Figure 4A and Supplementary Figure 6). In addition, Co-IP assays showed that BRD7 could interact with c-Myc in HEK293T or HCT116 cells (Figures 4B,C and Supplementary Figure 7). To further determine the structural basis of the protein interaction between BRD7 and c-Myc, we constructed domain-truncated mutants of BRD7 and c-Myc (Figures 4D,G) and then further confirmed the interaction between different domains of BRD7 and c-Myc. The Co-IP results showed that BRD7 interacts with c-Myc in a manner dependent on N-terminus of BRD7 and the CP-domain of c-Myc (Figures 4E,F,H,I).

### Restoring c-Myc Levels Reverses the Effect of BRD7 Knockdown on the Inhibition of Cell Proliferation and Cell Cycle Progression in Colorectal Cancer Cells

**FIGURE 5:**
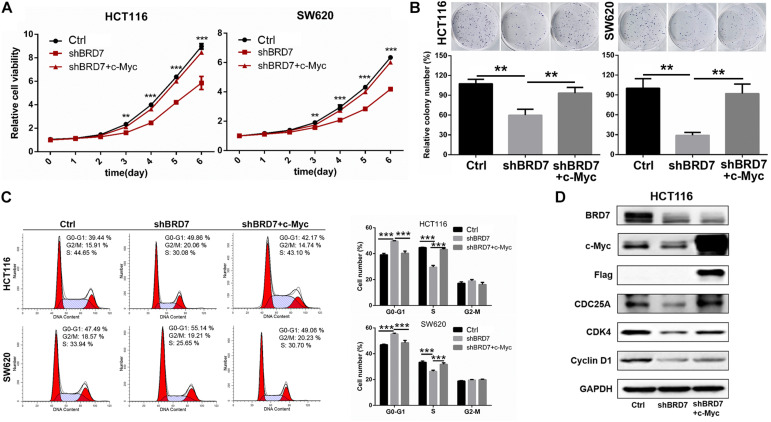
Restoring the expression of c-Myc reverses BRD7 knockdown-induced effects on cell proliferation and cell cycle progression in colorectal cancer (CRC) cells. **(A)** The effect of restoring c-Myc expression on BRD7 knockdown-induced cell proliferation inhibition, **(B)** cell colony formation, and **(C)** cell cycle G1/S phase arrest. **(D)** Western blotting confirmed the key molecules regulated by the BRD7/c-Myc axis. The results are presented as the mean ± SD. **P < 0.01, ***P < 0.001.

To further confirm our hypothesis that BRD7 promotes cell proliferation through stabilizing c-Myc protein, we investigated the effect of restored expression of c-Myc on BRD7 knockdown-induced cell proliferation inhibition and cell cycle arrest. CCK-8 and colony formation assays showed that the restoration of c-Myc levels could significantly rescue the cell viability and colony formation inhibition induced by BRD7 knockdown in HCT116 and SW620 cell lines when compared with that in the control groups (Figures 5A,B). Moreover, the flow cytometry results revealed that restoring c-Myc expression could markedly reverse the inhibitory effect of BRD7 knockdown on G1/S cell cycle arrest (Figure 5C). Increasing evidence has shown that CDC25A, CDK4, and cyclin D1, as c-Myc downstream targets, play crucial roles in cell cycle progression, especially in the G1/S phase transition (Macdonald and Bennett, 1999; Bouillez et al., 2016). Therefore, we detected the expression of some critical molecules involved in the G1/S phase transition of the cell cycle, and the immunoblotting results showed that the protein expression of CDC25A, CDK4, and cyclin D1 was significantly downregulated in BRD7 knockdown HCT116 cells compared with control cells, while the restoration of c-Myc expression markedly rescued the levels of CDC25A and CDK4 in BRD7 knockdown HCT116 cells (Figure 5D). Taken together, BRD7 promotes cell proliferation and cell cycle G1/S transition at least partially by stabilizing c-Myc protein.

### Targeting c-Myc Attenuates the Effect of BRD7 on Tumor Growth *in vivo*

**FIGURE 6:**
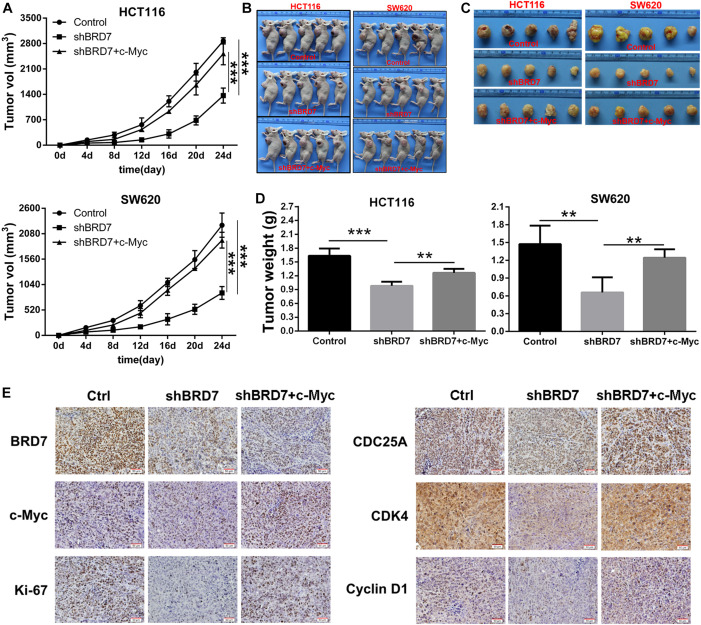
Restoring c-Myc expression reverses BRD7 silencing-induced inhibition of tumor growth. **(A)** Growth curve of HCT116 and SW620 xenograft tumors from nude mice. **(B)** Images of the HCT116 and SW620 xenograft tumors from nude mice. **(C)** Tumor images (*N* = 5). **(D)** Tumor weight quantification (*N* = 5). **(E)** Immunohistochemistry (IHC) confirmed the expression of key molecules downstream of the BRD7/c-Myc axis *in vivo*. Scale bar: 50 μm. The results are presented as the mean ± SD. **P < 0.01, ***P < 0.001.

To further confirm the functional roles of the BRD7/c-Myc axis in tumor growth and progression, we established a xenograft model in nude mice with BRD7 knockdown and BRD7 knockdown with simultaneous c-Myc restoration in HCT116 and SW620 cells. We found that BRD7 knockdown significantly inhibited tumor growth in both HCT116 and SW620 cells (Figure 6A). In addition, the tumor weight was markedly reduced in the BRD7 knockdown group compared with the control group (Figures 6B–D), while restoring c-Myc expression reversed BRD7 knockdown-mediated tumor growth inhibition in both HCT116 and SW620 cells (Figures 6A–D). These results demonstrated that BRD7 promotes CRC tumor growth at least partially by targeting c-Myc stability *in vivo*. In addition, we further determined the expression of key molecules downstream of the BRD7/c-Myc axis, and the results showed that BRD7 knockdown significantly downregulated the expression of c-Myc, CDC25A, CDK4, and cyclin D1 (Figure 6E), while restoring c-Myc expression attenuated BRD7 knockdown-induced downregulation of CDC25A, CDK4, and cyclin D1 (Figure 6E). Taken together, our findings demonstrated that targeting c-Myc attenuates the effect of BRD7 on tumor growth and cell cycle progression *in vivo*.

### A High Level of BRD7 Is Positively Associated With c-Myc Expression, Clinical Stage, and Poor Prognosis in Colorectal Cancer Patients

**FIGURE 7:**
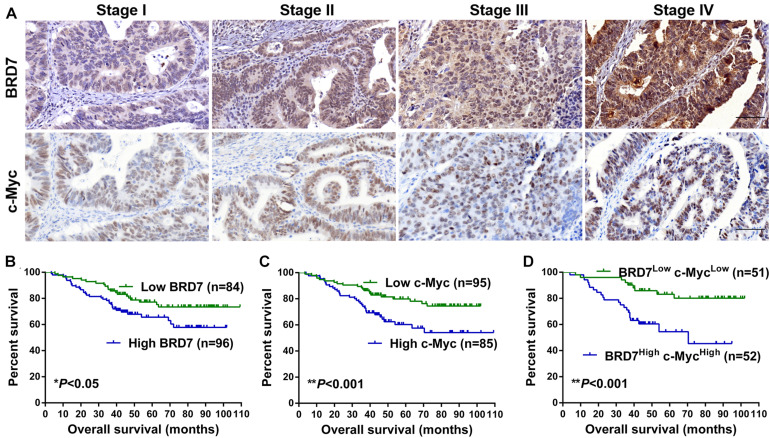
The expression and clinical significance of BRD7 and c-Myc in colorectal cancer (CRC) patients. **(A)** The expression of BRD7 and c-Myc protein in patients with different clinical stages of CRC, as detected by immunohistochemistry (IHC). Scale bar: 100 μm. **(B–D)** Kaplan–Meier analysis was used to plot the overall survival (OS) curve of CRC patients according to the expression of BRD7 and c-Myc.

To explore the clinical significance of the BRD7/c-Myc axis, the expression of BRD7 and c-Myc was assessed by IHC staining in CRC tissues derived from 180 patients with different clinical stages (Table 1). Most patients exhibited enhanced expression of BRD7, which was significantly associated with differentiation grade (Table 1). Additionally, the expression of BRD7 and c-Myc was markedly associated with the clinical stage of CRC patients (Table 1 and Figure 7A). The expression of c-Myc was significantly positively correlated with the expression of BRD7 in CRC patients (*P* = 0.006, *r* = 0.205, Table 2). Moreover, the survival analysis showed that CRC patients with high levels of BRD7 or c-Myc expression had poor prognoses (Figures 7B,C). In addition, CRC patients with high expression of both BRD7 and c-Myc had unfavorable prognoses (Figure 7D). These findings indicated that both BRD7 and c-Myc play critical roles in CRC progression and that the BRD7/c-Myc axis could serve as a potential biomarker and therapeutic target for the early diagnosis and treatment of CRC patients.

**TABLE 2:** BRD7 expression were positively correlated with c-Myc expression in CRC patients (*N* = 180).

		BRD7 expression			

		High	Low	*P*	*r*
**c-Myc expression**					
	High	52	33	0.006	0.205
	Low	44	51		

*CRC, colorectal cancer.*

## Discussion

Increasing evidence shows that BRD7 is widely downregulated and contributes to tumorigenesis in many malignancies (Chiu et al., 2014; Gao et al., 2016; Yu et al., 2016; Hu et al., 2019). BRD7 functions as a tumor suppressor, and low expression of BRD7 serves as an independent factor for prognosis and is positively associated with the clinical stage of cancer progression (Liu et al., 2016, 2018). In our previous study, we investigated the impact of BRD7 knockout (BRD7^–/–^) on DSS-induced colitis and found that BRD7 plays an anti-inflammatory role during early acute colitis in mice (Zhao et al., 2017); the results showed that DAI was significantly increased in DSS-induced acute colitis in BRD7^–/–^ mice from 1 to 5 days, consistent with its tumor-suppressive function in carcinogenesis. In this study, we further employed a well-established AOM/DSS model of CAC in BRD7 knockout mice. Unexpectedly, DAI was markedly reduced in the late stage of AOM/DSS-induced CRC in BRD7^–/–^ mice, and 70% of BRD7^+/+^ mice developed CAC, while there were no tumors in the BRD7^–/–^ group on day 126, indicating that BRD7 plays a pro-inflammatory role and potentially plays an oncogenic role in the late stage of AOM/DSS-induced CRC in mice. Moreover, BRD7 was markedly upregulated in CRC cells, and BRD7 knockdown inhibited cell proliferation, colony formation, and G1/S phase progression in two CRC cell lines. The *in vivo* assays also revealed that BRD7 promotes cell proliferation and tumor growth through facilitating the G1/S phase transition of the cell cycle. All of these findings demonstrate that BRD7 could significantly inhibit the occurrence of early inflammation and exert a tumor suppressor role but significantly promote the process of colorectal carcinogenesis in the late stage. It is suggested that BRD7 has a significant “functional duality” in different stages of the occurrence and development of CRC, and it is different from the role of tumor inhibition in other tumors, which is also a novel finding for BRD7 functions. Actually, more and more genes with dual functions as a tumor suppressor or an oncogene in cancer development and progression have been identified, such as Yin Yang 1 (YY1) (Sarvagalla et al., 2019) and TGF-β (Xu J. et al., 2015), and what functions they play may depend on the type of tumors as well as the genotype and expression of some critical genes in tumor cells. For example, YY1 was identified as a critical transcription factor, which has been implicated as a major driver of many cancers, while recent reports indicated that YY1 also functions as a tumor suppressor, and the mechanism by which YY1 brings out opposing outcome in tumor growth vs. suppression is not completely clear (Sarvagalla et al., 2019). Therefore, it could be understood that BRD7 has dual functions as a tumor suppressor and an oncogene.

BRD7 belongs to the bromodomain-containing protein family; it is primarily localized in the cell nucleus (Zhou et al., 2006; Wang et al., 2014) and serves as a crucial transcriptional regulatory factor (Burrows et al., 2010; Xu et al., 2016; Liu et al., 2017). Accumulating evidence has revealed that BRD7 is involved in cell cycle arrest via regulation of Ras/Raf/MEK/ERK, Rb/E2F, and AKT signaling in tumorigenesis and cancer progression (Zhou et al., 2004; Liu et al., 2016; Zhang Q. et al., 2016). In the present study, our results show that c-Myc downstream targets CDC25A, CDK4, and cyclin D1 are significantly downregulated in BRD7 knockdown CRC cells. Furthermore, the restoration of c-Myc expression markedly reversed the suppressive effects of BRD7 knockdown on the proliferation of CRC cell lines *in vitro* and *in vivo*. These findings demonstrate that BRD7 exerts its oncogenic role at least partially through c-Myc signaling and that the BRD7/c-Myc axis plays an important role in CRC progression.

c-Myc, which serves as a crucial transcription factor, regulates over 15% of genes of the human transcriptome (Dang et al., 2006; Kress et al., 2015) and is ectopically expressed or hyperactivated in a wide variety of malignancies (Wolf et al., 2015; Xiao et al., 2017; Jung et al., 2018; Li et al., 2019), including CRC; and c-Myc knockdown significantly inhibited cell proliferation and tumor growth (Niu et al., 2015; Li et al., 2017; Wang et al., 2018), revealing that c-Myc could be a broadly applicable and promising therapeutic target. Unfortunately, although the functional role and mechanism of c-Myc is well-defined in promoting cell proliferation in many cancers, no anti-cancer strategies that directly target c-Myc protein are available for clinical cancer treatment (Koh et al., 2016), owing to its “undruggable” protein structure (Bachmann and Geerts, 2018; Yoshida, 2018; Wang et al., 2019). Interestingly, we found that BRD7 knockdown significantly downregulates c-Myc protein levels and reduces the half-life of c-Myc protein. Mechanistically, BRD7 interacts with c-Myc protein through the N-terminus of BRD7 and the CP domain of c-Myc and therefore potentially competitively inhibits T58 phosphorylation of c-Myc triggered by GSK3β, whose activity is regulated by PI3K/Akt signaling. Then dephosphorylation of T58 stabilizes c-Myc by inhibiting T58 phosphorylation-dependent ubiquitination and degradation through the proteasome system (Hann, 2006; Sun et al., 2015). In addition, FBW7 was reported to be the E3 ubiquitin ligase involved in this process of T58 dephosphorylation stabilizing c-Myc protein (Welcker et al., 2004; Yada et al., 2004). Therefore, we speculated that FBW7 might be the E3 ubiquitin ligase involved in the stabilization of c-Myc mediated by BRD7; the detail mechanism needs to be further confirmed in the future work. Importantly, both BRD7 and c-Myc are significantly upregulated in CRC patients, and the expression of BRD7 is positively associated with the expression of c-Myc protein; high expression of these proteins indicates advanced clinical stage and poor prognosis in CRC patients. Therefore, based on our research, the development of anti-tumor small molecule inhibitors targeting BRD7/c-Myc axis could be an attractive approach in solving the problem of c-Myc “undruggable” and the clinical targeted therapy of CRC.

In summary, our study demonstrated that BRD7 exerts an oncogenic role and promotes CRC progression through inhibiting ubiquitin–proteasome-mediated degradation of the c-Myc protein. Both BRD7 and c-Myc are significantly upregulated in CRC patients, and the expression of BRD7 is positively associated with high levels of c-Myc protein; the high expression of these proteins indicates advanced clinical stage and poor prognosis in CRC patients. More importantly, a potent and selective inhibitor for BRD7 was reported recently (Clark et al., 2015), which provides a promising therapeutic option for BRD7-driven cancers, revealing that targeting the BRD7/c-Myc axis could be an attractive approach for the treatment of c-Myc-driven cancers.

## Data Availability Statement

The original contributions presented in the study are included in the article/Supplementary Material, further inquiries can be directed to the corresponding author/s.

## Ethics Statement

The animal study was reviewed and approved by the Institutional Animal Care and Use Committee (IACUC) of Central South University.

## Author Contributions

MZ designed the project and supervised the research. RZ, YL, CW, ML, WN, and YW performed the experiments. WW, SF, JY, HL, and YX collected the samples. RZ and YL wrote the manuscript. MZ, RZ, YL, YX, HL, ZZ, WX, XL, and GL reviewed and polished the manuscript. RZ, YL, and SF analyzed the data. All authors read and approved the final manuscript.

## Conflict of Interest

The authors declare that the research was conducted in the absence of any commercial or financial relationships that could be construed as a potential conflict of interest.

## Abbreviations

AOM: azoxymethane
BRD7: bromodomain-containing protein 7
bHLHZ: basic helix-loop-helix leucine zipper
CRC: colorectal cancer
CAC: colitis-associated carcinogenesis
CCK-8: Cell Counting Kit-8
CHX: cycloheximide
Co-IP: co-immunoprecipitation
DAI: disease activity index
DSS: dextran sodium sulfate
DMEM: Dulbecco’s modified Eagle medium
EdU: 5-ethynyl-2’-deoxyuridine
FBS: fetal bovine serum
FL: full length
H&E: hematoxylin and eosin
IF: immunofluorescence
IHC: immunohistochemistry
OS: overall survival
ORF: open reading frame
PVDF: polyvinylidene fluoride
qPCR: quantitative real-time PCR
RT-PCR: reverse transcription PCR
SDS-PAGE: sodium dodecyl sulfate–polyacrylamide gel
TAD: transcription activation domain.
