# Supplementary material for: BRD7 Promotes Cell Proliferation and Tumor Growth Through Stabilization of c-Myc in Colorectal Cancer
Source: Front Cell Dev Biol. 2021 May 24;9:659392. doi: 10.3389/fcell.2021.659392 (PMC8181413; doi:10.3389/fcell.2021.659392)
Supplement: Supplementary file 1 [file Data_Sheet_1.docx]

Supplementary Material

**
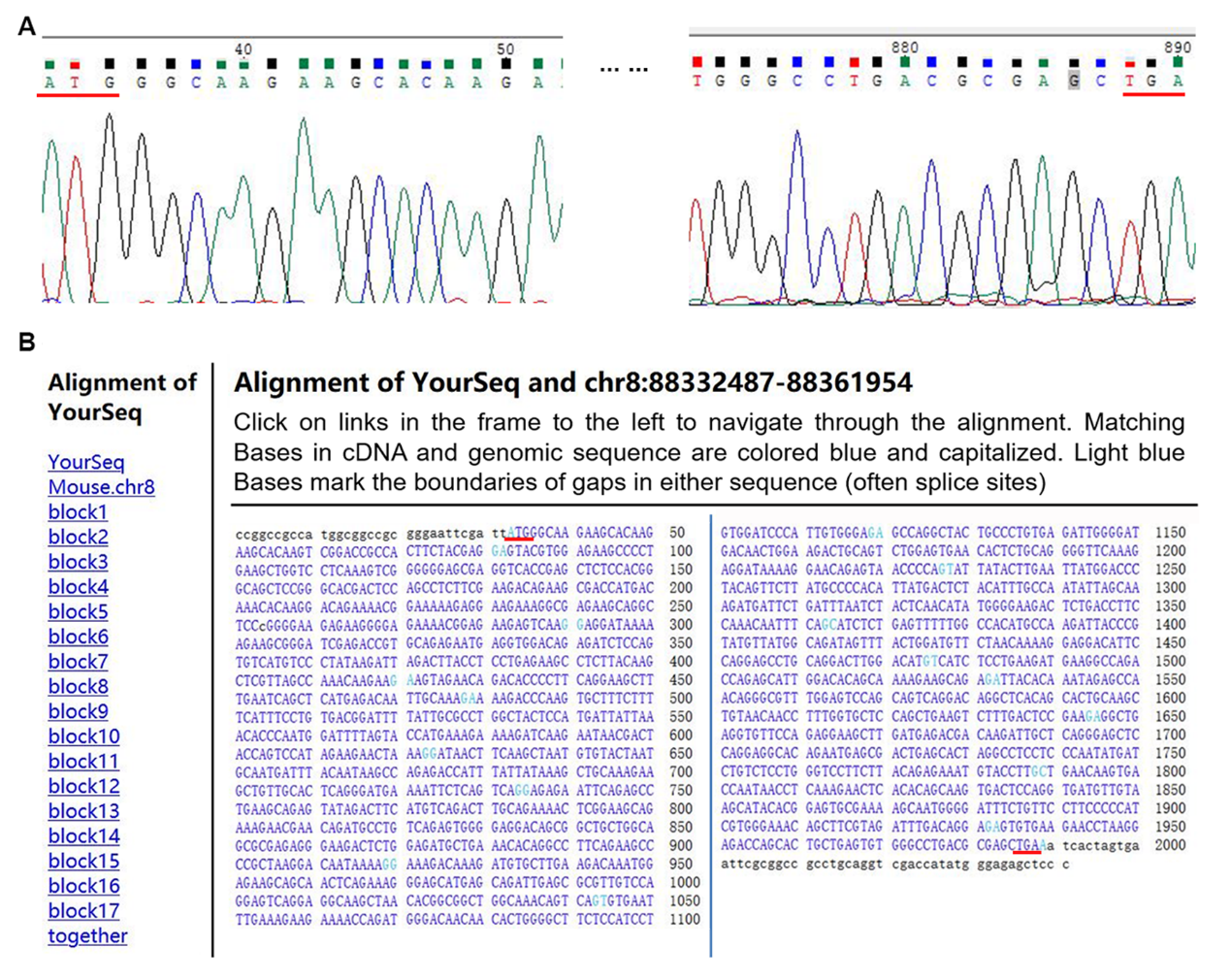
**

**Supplementary Figure 1.**The data of sequencing from colorectal cancer tissue of AOM/DSS induced BRD7^+/+^ mice. The RNA was extracted from colorectal cancer tissue of AOM/DSS induced BRD7 ^+/+^ mice, and then the reverse transcription and PCR were performed. The full-length BRD7 sequence was cloned into TA vector for sequencing analysis. **(A)** The display of head and tail peaks of sequencing results. **(B)** The sequencing data was input into the database <http://genome.ucsc.edu/> for comparison, and it was a perfect match with chromosome 8 of mouse BRD7.


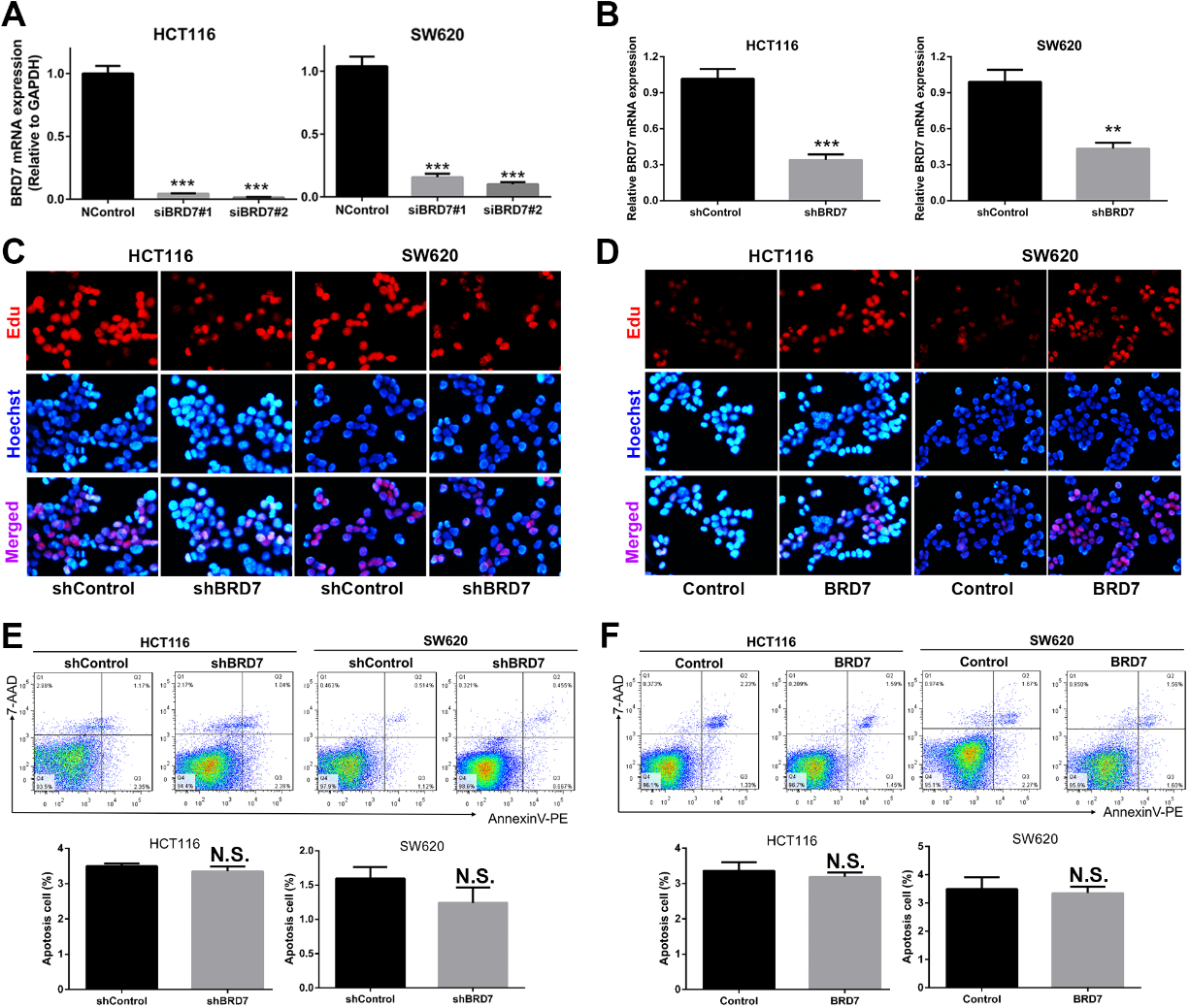


**Supplementary Figure 2.** The effect of BRD7 stable knockdown or overexpression on cell proliferation and apoptosis. **(A)** qRT-PCR confirmed the interference efficiency of siRNAs on endogenous BRD7 mRNA levels in HCT116 and SW620 cells. **(B)** qRT-PCR confirmed the expression of endogenous BRD7 mRNA levels in HCT116 and SW620 cells with stable BRD7 knockdown. **(C)** and **(D)** The effects of BRD7 knockdown or overexpression on cell cycle progression, as determined by EdU incorporation assays, respectively. **(E)** and **(F)** The effect of BRD7 stable knockdown or overexpression on cell apoptosis, as determined by flow cytometry. The results are presented as mean ± SD. N.S.=no significant, **p<0.01, ***p<0.001.


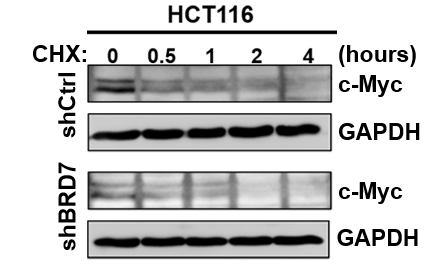


**Supplementary Figure 3.** The effect of BRD7 knockdown on the half-life of c-Myc protein in CHX-treated HCT116 cells at different time points (0, 0.5, 1, 2 and 4 hours). CHX: cycloheximide.


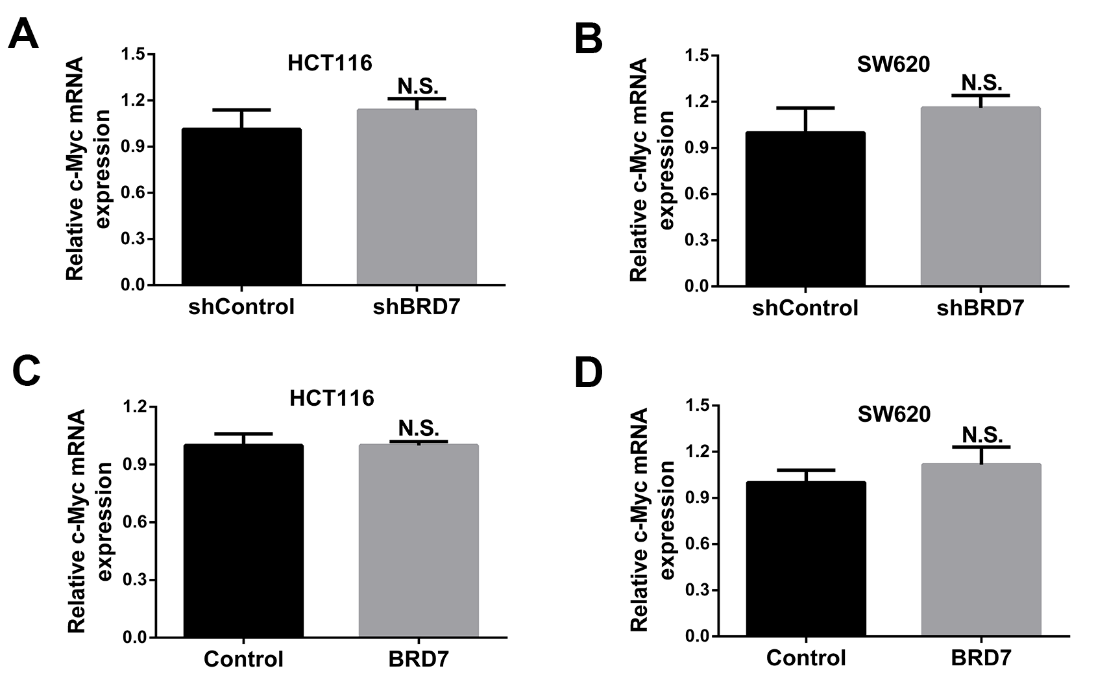


**Supplementary Figure 4.** The effect of BRD7 knockdown or overexpression on c-Myc mRNA levels. qRT-PCR confirmed the effect of BRD7 knockdown **(A, B)** or overexpression **(C, D)** on c-Myc mRNA levels in HCT116 and SW620 cell lines. The results are presented as mean ± SD. N.S.=no significant.

**
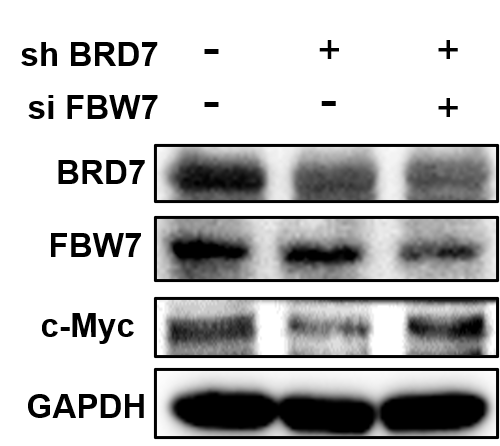
**

**Supplementary Figure 5.** FBW7 mediates the degradation of c-Myc by BRD7 in HCT116 cells.

**
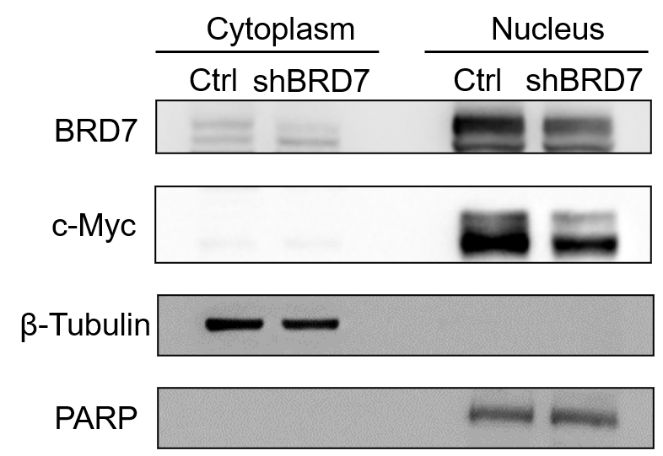
**

**Supplementary Figure 6.** The nuclear and cytoplasmic fraction was separated and used for the protein detection of BRD7 and c-Myc by western blot analysis.


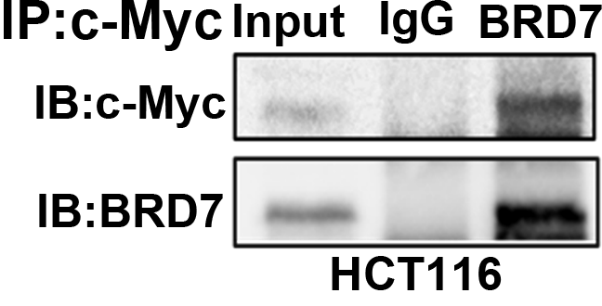


**Supplementary Figure 7.** BRD7 interacts with c-Myc in HCT116 cells. Co-IP confirmed the interaction between BRD7 and c-Myc in HCT116 cells.
